# A Curious Anecdote of the Rev. Sidney Smith

**Published:** 1855-07

**Authors:** 


We find the following good one going the rounds in the daily press. It is an "awful commentary" on what a lady friend characterized the other day as "*big, fat words.*"

*A Curious Anecdote of the Rev. Sidney Smith.—A decided sell.*—Lady Cubebs had a great passion for the garden and the hot-house, and when she got hold of a celebrity like the Reverend Sidney, was sure to dilate upon her favorite subject. Her Geraniums, her Auriculas, her Dahlias, her Carnations, her Acacias, her Lillia Regia, her Ranunculus, her Marygolds, her Peonies, her Rhododendron Procumbens, Mossy Pompone and Rose pubescens, were discussed with all the flow of hot-house rhetoric. "My Lady,"

asked the Reverend wit, "did you ever have a Psoriasis Septennis?" Oh yes—a most b-e-a-u-tiful one. *I gave it to the Archbishop of Canterbury.* Dear man!—and it came out *so* in the spring!"

